# Dexamethasone Rapidly Increases GABA Release in the Dorsal Motor Nucleus of the Vagus via Retrograde Messenger-Mediated Enhancement of TRPV1 Activity

**DOI:** 10.1371/journal.pone.0070505

**Published:** 2013-07-30

**Authors:** Andrei V. Derbenev, Bret N. Smith

**Affiliations:** 1 Department of Physiology, University of Kentucky College of Medicine, Lexington, Kentucky, United States of America; 2 Department of Cell and Molecular Biology, Tulane University, New Orleans, Louisiana, United States of America; 3 Department of Physiology, Tulane University School of Medicine, New Orleans, Louisiana, United States of America; University of Cincinnatti, United States of America

## Abstract

Glucocorticoids influence vagal parasympathetic output to the viscera via mechanisms that include modulation of neural circuitry in the dorsal vagal complex, a principal autonomic regulatory center. Glucocorticoids can modulate synaptic neurotransmitter release elsewhere in the brain by inducing release of retrograde signalling molecules. We tested the hypothesis that the glucocorticoid agonist dexamethasone (DEX) modulates GABA release in the rat dorsal motor nucleus of the vagus (DMV). Whole-cell patch-clamp recordings revealed that DEX (1-10 µM) rapidly (i.e. within three minutes) increased the frequency of tetrodotoxin-resistant, miniature IPSCs (mIPSCs) in 67% of DMV neurons recorded in acutely prepared slices. Glutamate-mediated mEPSCs were also enhanced by DEX (10 µM), and blockade of ionotropic glutamate receptors reduced the DEX effect on mIPSC frequency. Antagonists of type I or II corticosteroid receptors blocked the effect of DEX on mIPSCs. The effect was mimicked by application of the membrane-impermeant BSA-conjugated DEX, and intracellular blockade of G protein function with GDP _β_S in the recorded cell prevented the effect of DEX. The enhancement of GABA release was blocked by the TRPV1 antagonists, 5’-iodoresiniferatoxin or capsazepine, but was not altered by the cannabinoid type 1 receptor antagonist AM251. The DEX effect was prevented by blocking fatty acid amide hydrolysis or by inhibiting anandamide transport, implicating involvement of the endocannabinoid system in the response. These findings indicate that DEX induces an enhancement of GABA release in the DMV, which is mediated by activation of TRPV1 receptors on afferent terminals. The effect is likely induced by anandamide or other ‘endovanilloid’, suggesting activation of a local retrograde signal originating from DMV neurons to enhance synaptic inhibition locally in response to glucocorticoids.

## Introduction

Parasympathetic autonomic control of most thoracic and abdominal viscera is accomplished by neurons in the medullary dorsal vagal complex. The dorsal vagal complex consists of area postrema, the nucleus tractus solitarius (NTS), and the dorsal motor nucleus of the vagus (DMV). Axons of preganglionic motor neurons in the DMV project throughout most of the gastrointestinal (GI) tract and other subdiaphragmatic viscera [1], as well as contributing to innervation of thoracic organs. Neurons of the DMV exhibit regular action potential firing [2,3]. This makes the neurons susceptible to small changes in membrane potential induced by synaptic currents. This is especially true for inhibitory GABAergic inputs, which have a large influence on DMV neuron activity and parasympathetic output to the viscera [4].

In addition to well-described feedback effects on the hypothalamo-pituitary axis [5–7], stress and glucocorticoid hormones have long been known to alter autonomic function by modulating central autonomic circuitry. Glucocorticoid receptors are located in the dorsal vagal complex [8], including both the NTS and DMV, suggesting actions on central parasympathetic circuits. Central infusion of the glucocorticoid agonist dexamethasone (DEX) increases food intake, body weight, and insulin output, and promotes insulin resistance in rats [9]. These effects are prevented by vagotomy, suggesting glucocorticoid-mediated modulation of central parasympathetic circuits. Indeed, glucocorticoid agonists appear to act within the vagal complex to rapidly alter various autonomic functions related to gastrointestinal and cardiovascular control [10–13], possibly via GABA receptor-dependent effects [13], but the cellular mechanisms underlying these responses are unknown.

Rapid glucocorticoid actions on neuroendocrine or autonomic output [13–15] suggest potentially non-genomic effects in central autonomic circuits. In hypothalamic neuroendocrine and preautonomic neurons, glucocorticoids act on putative membrane-associated receptors in to rapidly stimulate the retrograde local release of endogenous cannabinoids and possibly other retrograde messengers, which in turn modulate afferent synaptic transmission [16–18]. Evidence for rapid cellular responses to activation of a somatic glucocorticoid receptor has also been shown in hippocampal cell cultures [19], and glucocorticoid receptors associated with neuronal membranes have been identified anatomically in the rat lateral amygdala [20].

Endocannabinoids released by glucocorticoid receptor activation or other means tend to regulate synaptic transmission by serving as retrograde messengers that are released from cell membranes and bind to receptors on afferent synaptic terminals [21–23]. One of these retrograde signaling molecules, anandamide, is synthesized in neurons of the vagal complex [24], and exogenously applied anandamide acts as an agonist at both cannabinoid type 1 (CB1) receptors and transient receptor potential vanilloid type 1 (TRPV1) on synaptic terminals to modulate inhibitory synaptic input to DMV neurons [25,26]. TRPV1 receptors are expressed in nodose ganglion cells [27] and are localized in primary viscerosensory afferent terminals in the DVC, where their activation modulates glutamate release onto NTS neurons [28,29]. Neurons in the NTS also express TRPV1 mRNA [30], and modulation of GABAergic synapses in the DMV by TRPV1 agonists is mediated in large part through heterosynaptic activation of glutamate release [25], suggesting that terminals of NTS neurons may also contain TPRV1 receptors. The effects of anandamide released endogenously after strong depolarization includes CB1 receptor activation [31], but the potential for glucocorticoid-mediated endocannabinoid release has never been established in the vagal complex. We therefore investigated the effects of glucocorticoid activation in the rat DMV, testing the hypothesis that glucocorticoids exert rapid effects via a retrograde messenger-mediated modulation of inhibitory synaptic input to DMV neurons.

## Methods

### Ethics statement

All procedures were performed on adult male Sprague-Dawley rats (Harlan, Indianapolis, IN) in accordance with NIH Guidelines for the care and use of animals in research and were approved by the University of Kentucky (IACUC numbers 01063M6006; 2008-0378) and Tulane University (IACUC number 0146-1-03-089) Animal Care and Use Committees.

### Brainstem slice preparation

Transverse brainstem slices were prepared from male Sprague-Dawley rats (Harlan), 4-8 weeks of age as previously described [25]. Rats were deeply anesthetized by halothane or isoflurane inhalation to effect and killed by rapid decapitation while anaesthetized. Brains were rapidly removed and immersed in ice-cold (0-4 ^o^C), oxygenated (95% O_2_-5% CO_2_) artificial cerebrospinal fluid (ACSF) containing (in mM): 124 NaCl, 3 KCl, 26 NaHCO_3_, 1.4 NaH_2_PO_4_, 11 mM glucose, 1.3 CaCl_2_, and 1.3 MgCl_2_, pH = 7.3-7.4, with an osmolality of 290-310 mOsm/kg. Transverse brainstem slices (350-400 µm) containing the DVC were made using a vibrating microtome (Vibratome Series 1000; Technical Products Intl., St. Louis, MO). Slices were transferred to a recording chamber mounted on a fixed stage under an upright microscope (Olympus BX51WI; Melville, NY).

### Whole-cell recording

Neurons in the DMV were visually targeted for whole-cell patch-clamp recording under a 40x water immersion objective using infrared differential interference contrast (IR–DIC) optics (Olympus). Recording pipettes (2-4 MΩ open tip resistance) were filled with a solution containing (in mM): 130 Cs^+^-gluconate, 1 NaCl, 5 EGTA, 10 HEPES, 1 MgCl_2_, 1 CaCl_2_, 3 KOH, 2-4 Mg-ATP; pH=7.2, adjusted with 5 M CsOH. Neurons were voltage-clamped in the presence of tetrodotoxin (TTX; 1 µM) at -10 mV or -70 mV to assess miniature IPSCs (mIPSCs) or mEPSCs, respectively. As previously reported [2,25,32–34], the large outward synaptic currents recorded at -10 mV exhibited rapid 10-90% (~1 ms) rise time, exponential decay, were blocked by GABA_A_ receptor antagonists (n=5), and reversed near the Cl^-^ eqilibrium potential (~-80 mV); they were therefore considered to be GABAergic mIPSCs. Inward currents recorded at a holding potential of -70 mV also exhibited a rapid rise time and exponential decay and were blocked by kynurenic acid (n=5); they were considered to be glutamatergic mEPSCs. Electrophysiological signals were low pass filtered at 2-5 kHz, recorded using an Axopatch 700B amplifier (Axon Instruments, Union City, CA) and pClamp 10.2 software (Axon Instruments). IPSC frequency and amplitude were analyzed offline using Clampfit 10.2 and MiniAnalysis (Synaptosoft, Decatur, GA).

### Drug application

All experiments were performed with TTX (1 µM; Tocris) in the ACSF to block action potential-dependent synaptic activity; kynurenic acid (1 mM; Sigma) was added in some experiments to block ionotropic glutamate receptors. Water-soluble dexamethasone (DEX; Sigma) was bath applied for 3-10 minutes at a final concentration of 1-10 µM. Bovine serum albumen (BSA)-conjugated dexamethasone (Steraloids Inc., Newport, RI) was dissolved in the ACSF and bath applied for 3-10 minutes at a final concentration of 10 µM. Inhibitors of anandamide uptake O-2093 (10 µM) or OMDM-2 (10 µM), and the selective TRPV1 receptor antagonists 5´-iodoresiniferatoxin (5’-iRFT; 1 µM; Tocris) or capsazepine (1 µM; CPZ; Tocris) were first dissolved in ethanol and diluted to final concentration in ACSF (final concentration of ethanol <0.01% by volume). The CB1 receptor antagonist AM251 (10-20 µM; Tocris), the inhibitor of fatty acid amide hydrolase (FAAH), URB-597 (10 µM; Sigma), and the glucocorticoid receptor antagonists mifepristone (1 µM; Sigma) and spironolactone (1 µM; Sigma) were dissolved in DMSO (final DMSO concentration <0.01% by volume). Reuptake blockers and receptor antagonists were bath applied for at least 10 min prior to agonist application. The nonhydrolyzable guanylyl nucleotide GDP-β-S (500 µM; Sigma-Aldrich) was included in the pipette solution in some experiments to prevent G protein-coupled receptor activity in the recorded neuron.

### Data analysis

The effects of DEX on mIPSC or mEPSC frequency and amplitude were analyzed within a recording using the intra-assay Kolmogorov-Smirnov test (i.e., K–S test; a nonparametric, distribution-free, goodness-of-fit test for probability distributions). Pooled results from responding neurons were analyzed using a paired two-tailed Student’s t-test. For all analyses, P<0.05 was considered significant. Values are reported as the mean ± SEM.

## Results

### Dexamethasone increased mIPSC frequency in DMV neurons

**Figure 1:**
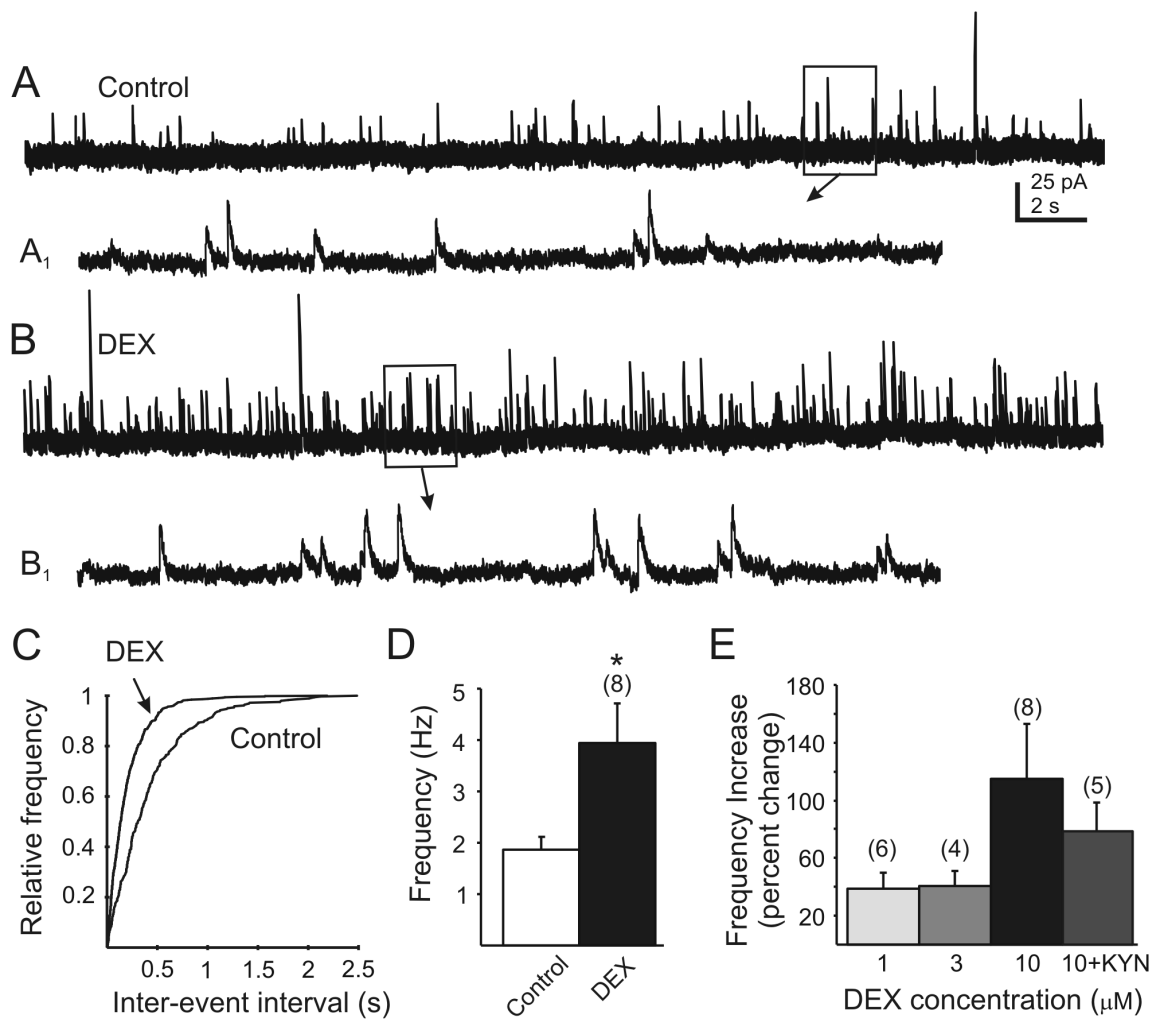
Dexamethasone increases mIPSC frequency in DMV neurons. **A**. Continuous recording of mIPSCs observed at holding potential of -10 mV in the presence of tetrodotoxin (TTX; 1 µM). **B**. Recording of mIPSCs in the same cell after application of DEX (10 µM). **A_1_** and **B_1_** are temporally-expanded, 2 s sections from boxed areas of traces A and B, respectively. Recording pipette contained 140 mM Cs^+^. **C**. Cumulative frequency plots of the inter-event interval distribution for mIPSCs before and after application of DEX for the recording in A and B. A significant effect of DEX was detected in this cell using the Kolmogorov-Smirnov (K–S) test (*P*<0.05). **D**. Change in mIPSC frequency observed 3 min after DEX application in DMV neurons that were determined to have responded to DEX by K–S test (n=8; p<0.05). **E**. The effect of DEX in responding cells as a function of concentration. The effect of the highest DEX concentration (10 µM) in the presence of kynurenic acid (KYN; 1 mM) is also shown. Numbers in parentheses above the bars indicate number of replicates; error bars indicate SE.

The effect of DEX on mIPSCs was examined in DMV neurons in the presence of TTX (1 µM). Neurons were voltage-clamped at -10 mV using Cs^+^ in the recording pipette to block voltage-dependent K^+^ channels and consequently improve quality of the voltage clamp [26,35]. Application of different concentrations of DEX (1-10 µM) resulted in a concentration-related increase in the frequency of mIPSCs (Figure 1E). All neurons were tested by the intra-assay K–S test to determine if DEX induced a significant change in mIPSC frequency within a recording, and neurons with a significant response were combined for further analysis. The effect of DEX was observed within 3 minutes of drug application. After application of 1 µM DEX, 6 out of 10 DMV cells (60%) responded with increase of frequency from 2.5±0.4 Hz (range 1.4-4) to 3.5±0.6 Hz (range 2-5.5; P<0.05; n=6). Application of 3 µM DEX resulted in an increase in mIPSC frequency in 4 out of 5 DMV neurons (80%), from 3.4±1 Hz (range 1.1–5.1 Hz) to 4.8±1.6 Hz (range 1.5-8 Hz; P<0.05; n=4). Bath application of 10 µM DEX significantly increased the frequency of mIPSCs in 8 out of 12 (67%) DMV cells (Figure 1). Under control conditions, the average of mIPSC frequency was 1.8±0.2 Hz (range 1-2.4 Hz). Mean mIPSC frequency was increased in the presence of DEX (10 µM) by 115%, to 3.9±0.8 Hz (range 2–8.3 Hz; P<0.05; n=8; Figure 1). The remaining neurons displayed no significant changes in mIPSC frequency in response to DEX. In the continuous presence of DEX, mIPSC frequency returned to levels similar to pre-application after 8-10 min (p>0.05 versus control). Overall, there was no effect of DEX on mIPSC amplitude (p>0.05), suggesting effects on mIPSC frequency were due to altered GABA release from presynaptic terminals.

### Dexamethasone actions are mediated by a spironolactone/mifepristone sensitive receptor

**Figure 2:**
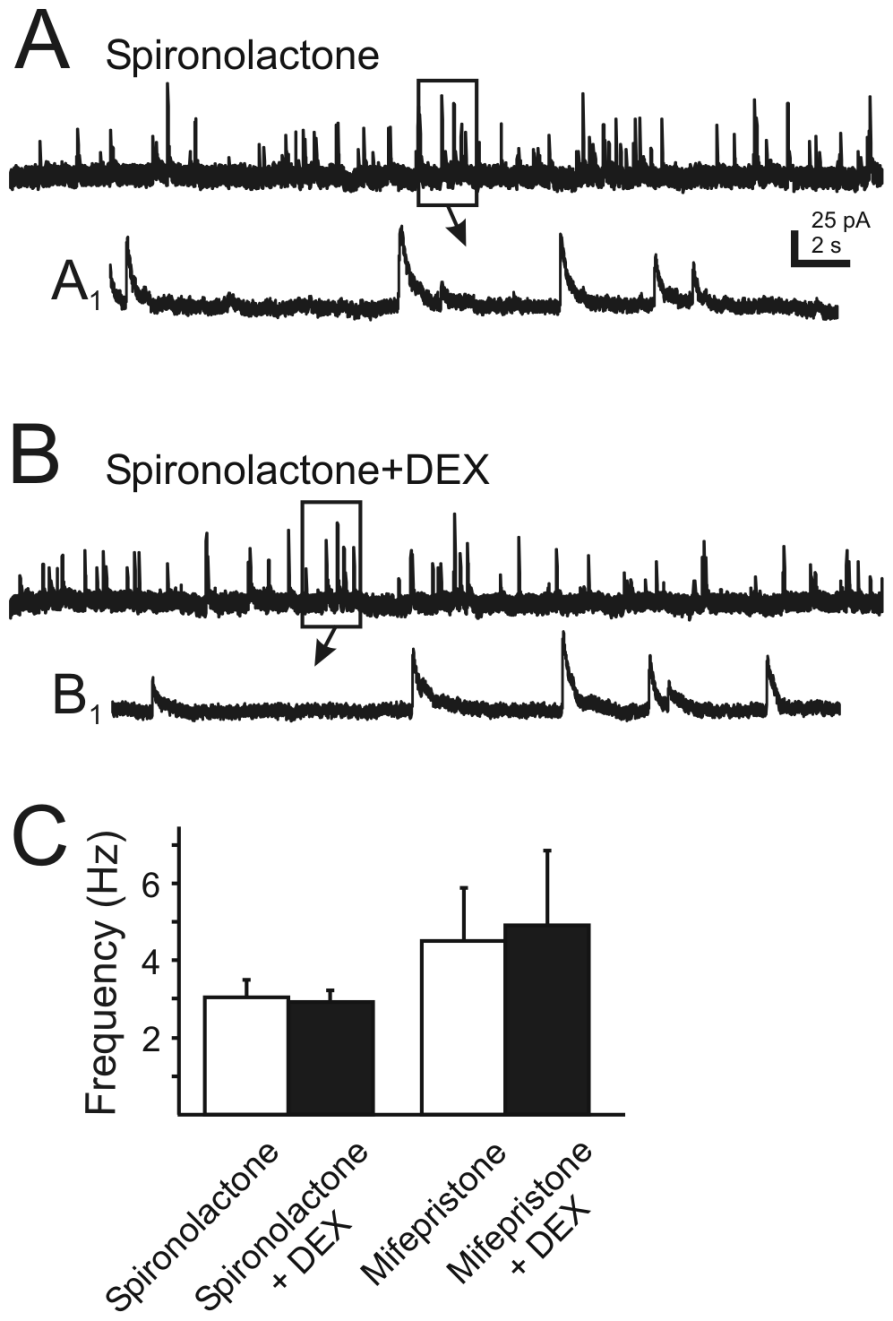
Rapid glucocorticoid effects are prevented by glucocorticoid receptor blockade. **A**. Continuous recordings of mIPSCs at a holding potential of -10 mV in the presence of the type I corticosteroid receptor antagonist, spironolactone (1 µM). **B**. The same cell after co-application of DEX (10 µM). **A_1_** and **B_1_** are temporally-expanded, 2 s sections from boxed areas of traces A and B (arrows). **C**. The DEX-induced increase in mIPSC frequency was never observed in the presence of type I and II corticosteroid receptors antagonists spironolactone (1 µM; n=9; p>0.05) or mifepristone (1 µM; n=9; p>0.05). Error bars indicate SE.

Antagonists of type I and II corticosteroid receptors blocked the DEX effect. Spironolactone (1 µM), an antagonist of type I corticosteroid receptors or mifepristone (1 µM), and antagonist of type II corticosteroid receptors was applied prior to and during DEX application. Neither spironolactone (1 µM) nor mifepristone (1 µM) alone significantly altered the frequency of mIPSCs, but each prevented the effect of DEX (10 µM; Figure 2). The frequency of mIPSCs was 3.0±0.4 Hz (range 1.1–5.2 Hz) in the presence of spironolactone (1 µM). After bath application of DEX (10 µM) in the presence of spironolactone (1 µM) the mean frequency of mIPSCs was 2.9±0.3 Hz (range 1.7–4.4 Hz; P>0.05; n=9). In another group of neurons, the frequency of mIPSCs was 4.5±1.4 Hz (range 1.5–13 Hz) in the presence of mifepristone (1 µM) and was 4.9±2 Hz (range 1.1–17.8 Hz) after addition of DEX (10 µM; P>0.05; n=9). The effect of DEX was blocked in the presence of antagonists to corticosteroid receptors.

### Dexamethasone actions are mediated by a membrane-associated receptor

**Figure 3:**
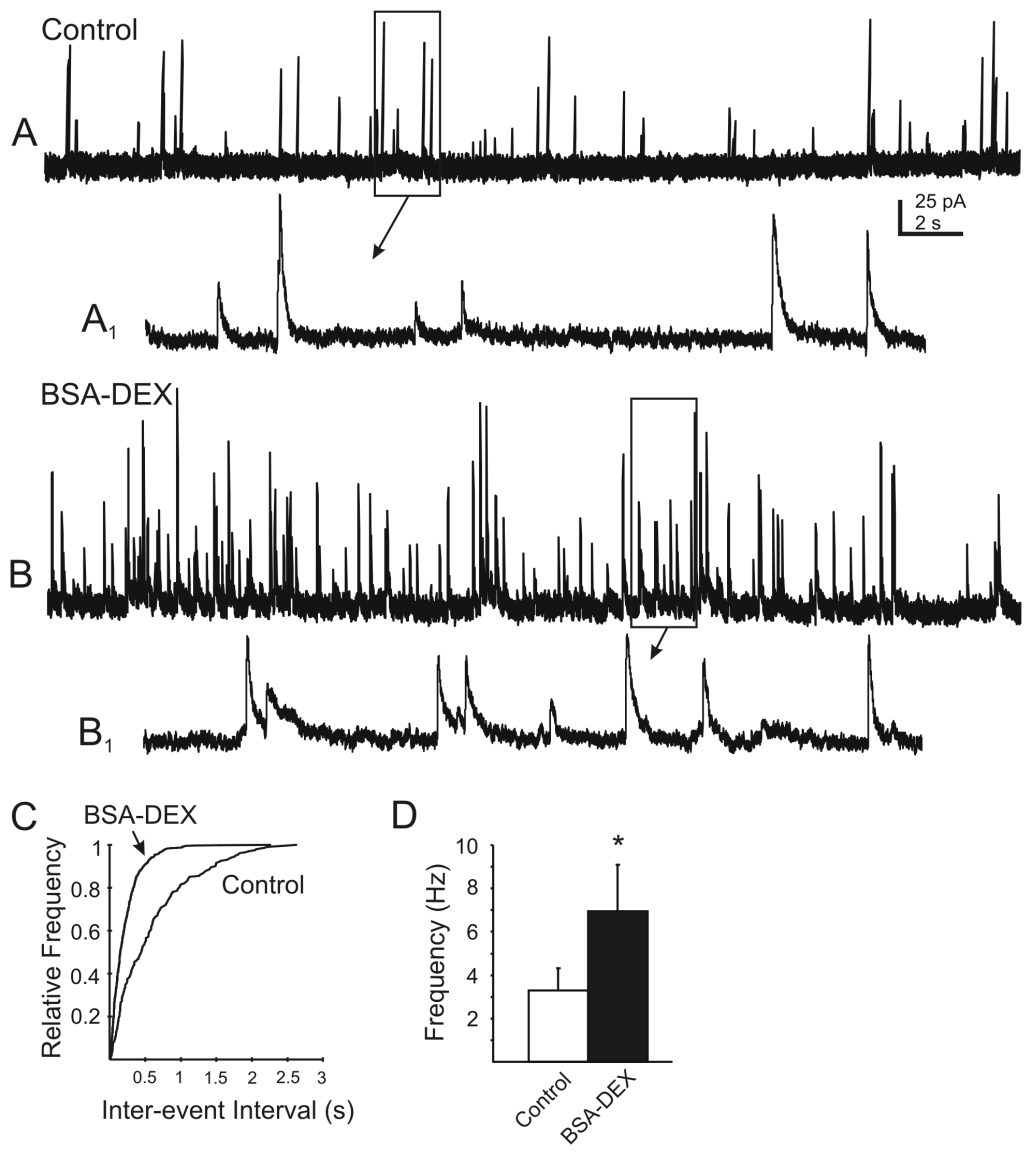
The membrane impermeant BSA-conjugated DEX enhanced mIPSC frequency, suggesting activation of a nonclassical membrane-associated glucocorticoid receptor. **A**. Continuous recording of mIPSCs observed at holding potential of -10 mV. **B**. mIPSCs recorded after application of a membrane-impermeable, BSA-conjugated DEX (10 µM). **A_1_** and **B_1_** are temporally-expanded, 2 s sections from boxed areas of traces A and B above (arrows). **C**. Cumulative plots of the inter-event interval distribution for mIPSCs before and after application of BSA-conjugated DEX from the recording in A and B (*P*<0.05; K–S test). **D**. Changes in mIPSC frequency in responsive DMV neurons after 3 min application of BSA-conjugated DEX (10 µM; n=7; p<0.05).

The rapid onset of the DEX effect on mIPSC frequency suggested a nongenomic mechanism of action, possibly mediated by a membrane-associated receptor [16]. Experiments were conducted to determine whether the DEX effect on GABA release could be prevented by reducing the ability of DEX to cross the cell membrane and act at intracellular receptors [36]. Bath application of the membrane-impermeable, BSA-conjugated DEX (10 µM) mimicked the effect of DEX on mIPSC frequency. Seven out of 11 cells (64%) responded to application of BSA-conjugated DEX (10 µM) with a significant increase in mIPSC frequency. The mIPSC frequency increased from 3.3±1 Hz (range 1.1–9 Hz) to 6.9±2.2 Hz (range 4-20 Hz; P<0.05; n=7; Figure 3) in the presence of BSA-conjugated DEX, with no significant change in mIPSC amplitude. These results suggested that the DEX effect occurred subsequent to activation of a receptor associated with the postsynaptic membrane and did not require binding to an intracellular receptor.

**Figure 4:**
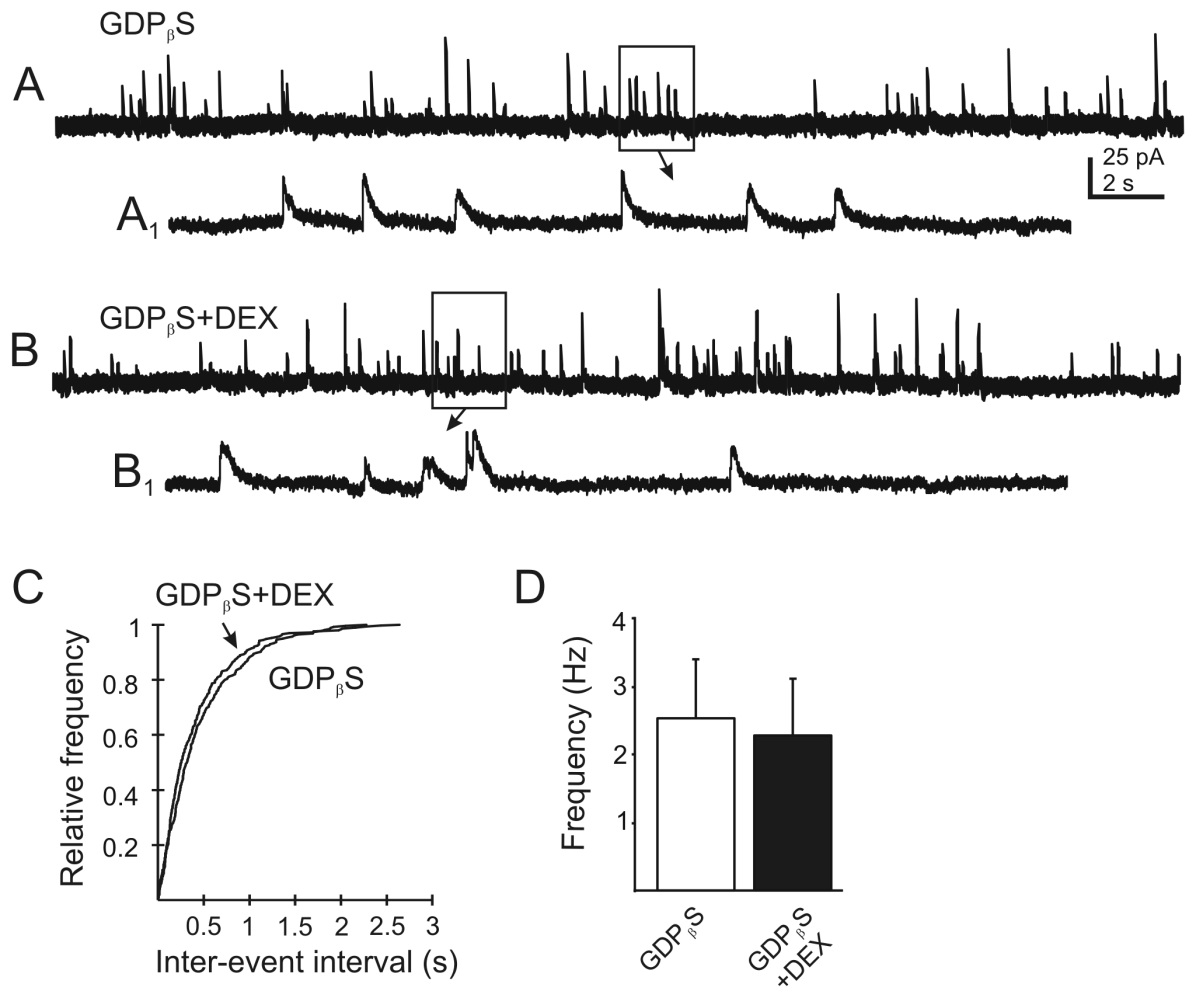
Blockade of G protein-mediated responses in the recorded neuron prevents the DEX-induced change in mIPSC frequency. **A**. Continuous recordings recording of mIPSCs after loading of the recorded cell with the G protein inhibitor, GDP _β_S (500 µM in the recording pipette). **B**. mIPSCs recorded in the same neuron after application of DEX (10 µM). **A_1_** and **B_1_** are temporally-expanded, 2 s sections from boxed areas of traces A and B (arrows). **C**. Cumulative plots of the inter-event interval distribution for mIPSCs in the GDP _β_S-loaded cell shown in A and B indicates no effect of DEX (*P*>0.05; K–S test). **D**. Graph of mean mIPSC frequency before and after DEX application in GDP _β_S-loaded neurons. No effect of DEX was observed in any neuron (n=13; P>0.05).

Rapid glucocorticoid effects in hypothalamic neuroendocrine neurons are mediated by a G protein-dependent, putative postsynaptic mechanism involving release of a retrograde signalling molecule to affect activity at synaptic terminals [16]. We tested for a similar mechanism in the DVM neurons. The G protein inhibitor, GDP _β_S (500 µM) was applied intracellularly via the recording pipette to inhibit G protein-mediated responses in the recorded neuron. Control experiments were performed to ascertain any time-dependent effect of GDP _β_S (500 µM) on mIPSC frequency in DMV neurons. The frequency of mIPSC in the first 5 min of obtaining the recording was 4±0.6 Hz (range 2.2–6 Hz). After 15 min, the frequency of mIPSC was 3.3±0.4 Hz (range 1.7-4.1 Hz; n=5). The GDP _β_S alone had no significant effect on mIPSC frequency (P>0.05) and amplitude was also unaffected. When GDP _β_S was included intracellularly in a different set of neurons, application of DEX (10 µM) was without effect on mIPSC frequency in any recorded neuron. In the presence of intracellular GDP _β_S, mIPSC frequency was 2.5±0.8 Hz (range 0.2–8 Hz). After application of the DEX, the mean frequency of mIPSCs was 2.2±.08 Hz (range 0.4–8.5 Hz; P>0.05; n=8; Figure 4). Because the GDP _β_S activity was likely restricted to the postsynaptic (i.e., recorded) cell, this suggested that the effect of DEX was dependent on G-protein-mediated activity in the recorded neuron.

### Inhibition of TRPV1 receptors prevented the effect of DEX

**Figure 5:**
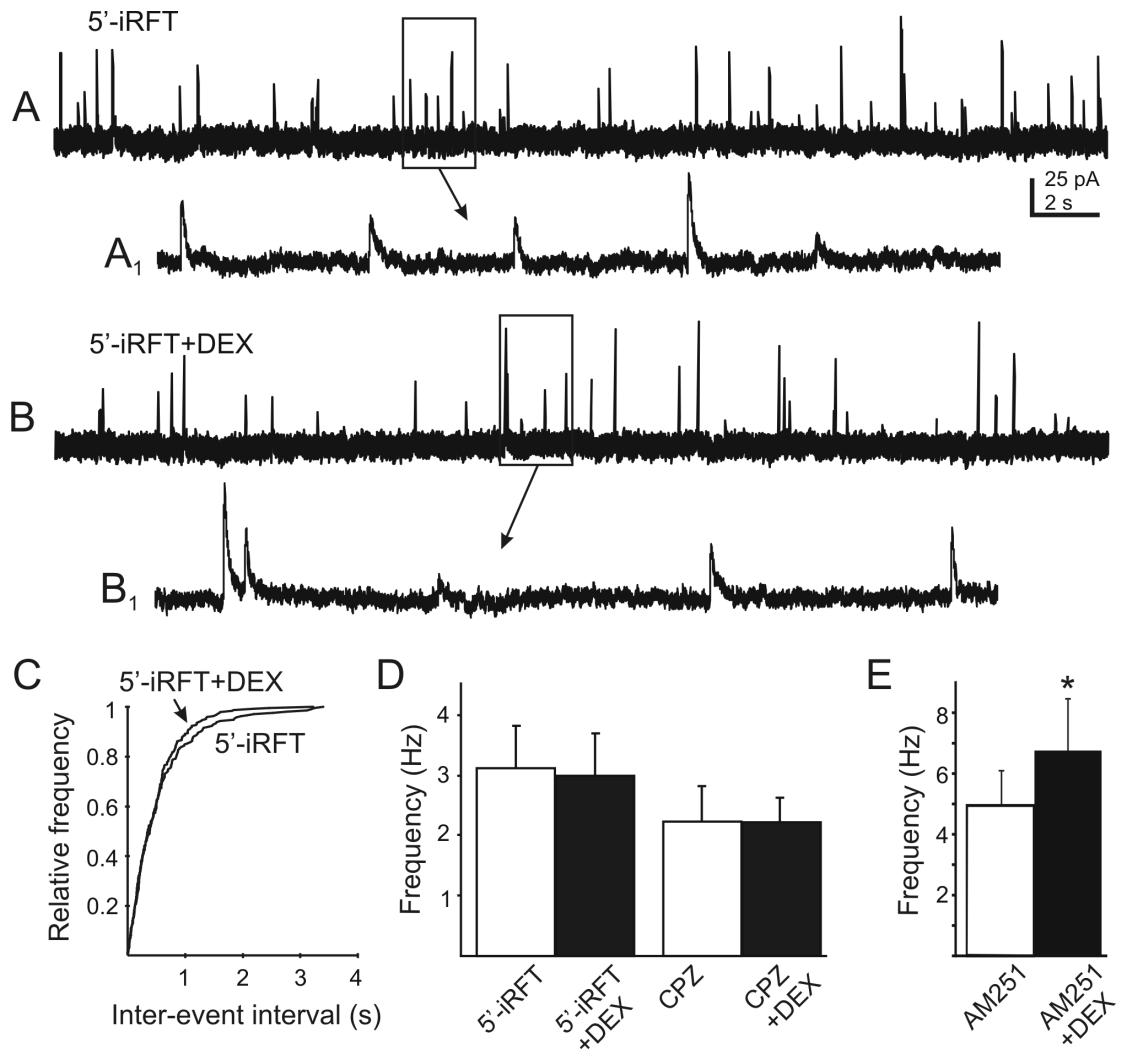
The glucocorticoid-induced increase in mIPSC frequency is blocked by TRPV1 antagonists. **A**. Continuous recordings of mIPSCs at a holding potential of -10 mV in the presence of the TRPV1 antagonist, 5’-iodoresiniferatoxin (5’-iRFT; 1 µM). **B**. The same cell after application of DEX (10 µM). **A_1_** and **B_1_** are temporally-expanded, 2 s sections from boxed areas of traces A and B (arrows). **C**. Cumulative frequency plots of data from the recording shown in A and B indicate no DEX-induced change in mIPSC inter-event interval (P>0.05; K–S test). **D**. The DEX-induced increase in mIPSC frequency was blocked with bath pre-application of 5’-iRFT (n=12; p>0.05) or capsazepine (CPZ; 10 µM; n=6; P>0.05). **E**. Application of DEX increased mIPSC frequency in the presence of the cannabinoid type 1 receptor antagonist, AM251 (20 µM; n=8; p<0.05).

Since blocking activity in the postsynaptic neuron prevented DEX-induced changes in mIPSC frequency, the DEX effects on presynaptic GABA release were hypothesized to be mediated by the actions of one or more retrograde messengers, consistent with previous reports in the hypothalamus [16,18,37,38]. Anandamide, a potential retrograde messenger in the DMV, is an agonist at both CB1 and TRPV1 receptors [36]. Activation of these receptors has opposing effects on GABA release in the DMV, with CB1 receptor activation inhibiting and TRPV1 receptor activation increasing mIPSC frequency [25,26]. Since the effect of DEX was to increase mIPSC frequency, and we previously showed that anandamide could activate TRPV1 receptors on GABAergic terminals [25], we tested the hypothesis that the DEX-induced increase in mIPSC frequency was mediated by TRPV1 receptors. To block TRPV1 activity, a potent and highly selective TRPV1 antagonist, 5'-iRFT (1 µM) was applied prior to and during DEX application. The antagonist alone had no effect on the frequency of mIPSCs, but completely prevented the effect of DEX (Figure 5A–D). The frequency of mIPSCs was 3.1±0.7 Hz (range 0.6–8 Hz) in the presence of 5'-iRFT and 3±0.7 (range 0.6–7.6 Hz) after bath application of DEX (10 µM) in the continued presence of 5'-iRFT (P>0.05; n = 12). Similarly, no change in mIPSC frequency was observed when DEX was applied in the presence of another TRPV1 antagonist, CPZ (10 µM; n=6; P>0.05; Figure 5D). Blockade of TRPV1 receptors prevented the increase in mIPSC frequency after DEX application, suggesting that DEX induced the release of an endogenous vanilloid receptor agonist.

We hypothesized that the facilitatory effect of DEX on GABA release onto DMV neurons might be negatively modulated by CB1 receptor-mediated inhibition of GABA release in the same terminals. Bath application of the CB1 receptor antagonist AM251 (20 µM) did not alter the DEX effect. Bath application of DEX (10 µM) in the presence of AM251 produced an increase in mIPSC frequency in 8 out of 13 neurons (62%), from 4.9±1.2 Hz (range 2-11; n=8) in AM251 to 6.9±1.7 Hz (range 3-14; n=8; P<0.05; Figure 5E) with the addition of DEX, similar to effects in the absence of the CB1 receptor antagonist. The effect of DEX on mIPSC frequency occurred independently of CB1 receptor-mediated activity.

### Dexamethasone effects on glutamate release

Enhancement of GABA release by bath-applied capsaicin is attenuated by ionotropic and metabotropic glutamate receptor antagonists, suggesting the TRPV1-dependent effect on mIPSC frequency is mediated in large part by a heterosynaptic, glutamate-dependent mechanism [25]. In the presence of kynurenic acid (1 mM) to block ionotropic glutamate receptors, DEX application (10 µM) resulted in an increase in mIPSC frequency of 79% (n=5; p<0.05; Figure 1E). This was notably less robust than the response in the absence of glutamate receptor blockers (i.e., 115%). Although the difference in response was not significant (p>0.05), the result suggested involvement of DEX-induced glutamate release in the GABAergic response to DEX. To determine if DEX caused a change in synaptic glutamate release, the effect of DEX on mEPSC frequency was tested in the presence of picrotoxin (100 µM), while voltage-clamping the recorded cell at -70 mV. DEX (10 µM) increased mEPSC frequency from 8.7±2.4 Hz (range 2.4-16.9 Hz) to 18.0±4.0 Hz (range 6.4-28.8 Hz; 127% increase; p<0.05), with no change in mEPSC amplitude (p>0.05). The effect on mEPSC frequency persisted throughout the DEX application (14.5 ±3.7 Hz at 10 min of continuous application). Similar to effects on GABA release, glutamate release was enhanced by DEX. Blockade of ionotropic glutamate receptors suppressed, but did not eliminate the effect of DEX on GABA release.

### Inhibition of anandamide metabolism and transport

**Figure 6:**
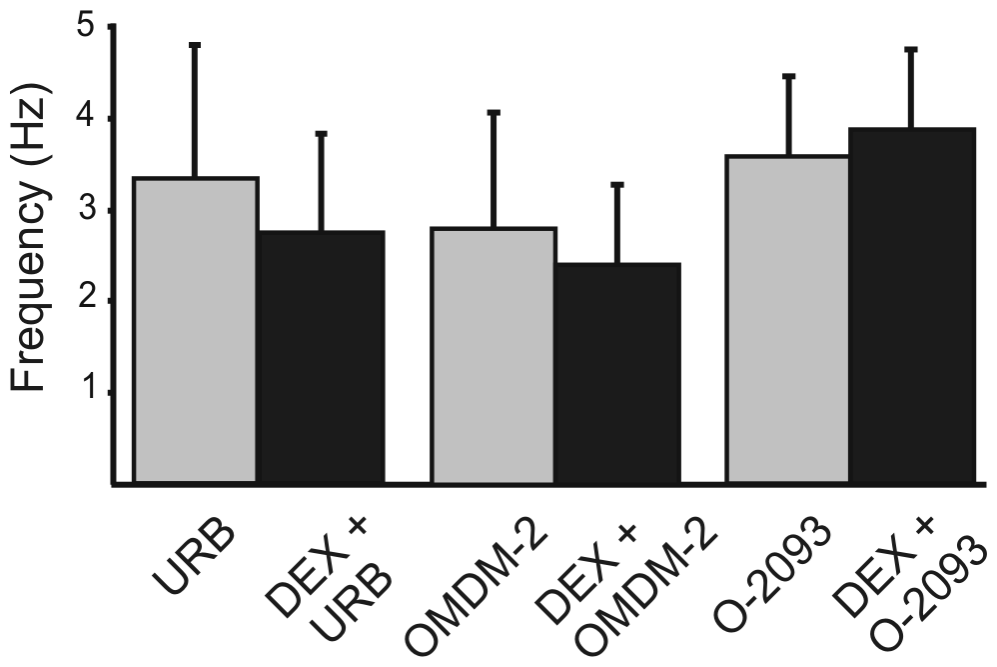
Blockade of anandamide metabolism or transport prevented the effect of DEX. The effect of DEX on mIPSC frequency was prevented in the presence of the fatty acid amide hydrolyse (FAAH) inhibitor, URB597 (10 µM; n=11; P>0.05). Blocking anandamide transport with OMDM-2 (10 µM; n=6; P>0.05) or O-2093 (10 µM; n=6; P>0.05) also prevented the effect of DEX on mIPSC frequency.

The endogenous cannabinoid, anandamide activates TRPV1 receptors, which leads to facilitation of neurotransmitter release in the DMV [25] and is therefore also an endogenous vanilloid agonist candidate. We examined the contribution of proteins involved in anandamide signalling on the effect of DEX on mIPSCs. The effect of DEX was tested in the presence of inhibitors of anandamide metabolism and transport. Bath application of URB-597 (10 µM), an inhibitor of FAAH, had no significant effect on mIPSC frequency (n=7; P>0.05). Following application of DEX (10 µM) in the presence of URB-597, no significant effect on mIPSC frequency was observed in any neuron (Figure 6). The frequency of mIPSCs was 3.4±1.5 Hz (range 0.5–15 Hz) in the presence of URB-597 and was 2.8±1.1 Hz (range 0.3-11.4; P>0.05; n=10) after DEX application. Inhibiting FAAH activity prevented the effect of DEX on mIPSC frequency.

The application of an anandamide transporter inhibitor, OMDM-2 also was without effect on mIPSC frequency (n=6; P>0.05). Application of DEX (10 µM) in the presence of OMDM-2 had no significant effect on the frequency of mIPSCs in any cell (2.4±0.9 Hz, range 0.4–6.1 Hz; n=6; P>0.05; Figure 6). Separate experiments performed using another anandamide transport inhibitor, O-2093 (10 µM) yielded similar results. The mean mIPSC frequency was 3.6±0.9 Hz (range 2.1-7.1 Hz) in the presence of O-2093 and was 3.9±0.9 Hz (range 2-7.2 Hz) after additional application of DEX (P>0.05; Figure 6). Blocking anandamide transport prevented the enhancement of mEPSC frequency by DEX.

## Discussion

Fast synaptic input to preganglionic parasympathetic motor neurons of the DMV is primarily GABAergic and glutamatergic [2,35]. Whereas glutamatergic input is thought to play a relatively minor role in regulating the ongoing activity of DMV neurons, GABA release is tonic and contributes significantly to activity levels of preganglionic DMV neurons [39,40]. A variety of neuromodulatory substances can affect GABAergic synaptic input to these cells, including candidate retrograde messengers like anandamide [25,26]. Rather than being stored and released from vesicles, anandamide and other endocannabinoids are instead synthesized in the cell membrane and released locally upon formation. They then act as retrograde signals that modify synaptic inputs, typically by binding to receptors on presynaptic terminals [41]. The release of endocannabinoids can be caused by a variety of different stimuli, including by activity-dependent mechanisms [22], neuromodulator-mediated G protein-coupled receptor activation [42], and rapid steroid actions [16]. The present findings, together with previous reports on activity-dependent endocannabinoid effects [31], indicate these mechanisms also affect GABA release in the DMV.

In the DMV, exogenously-applied anandamide binds to both CB1and TRPV1 receptors located on presynaptic axon terminals, where their activation leads to a mild suppression or profound enhancement, respectively, of neurotransmitter release [25,26]. Anandamide and several other endocannabinoid ligands are also classified as ‘endovanilloids’, based on their ability to activate TRPV1 receptors [36]. In fact, transient anandamide application principally acts at TRPV1 receptors on synaptic terminals in the DMV to potently increase GABA release, an effect that involved a heterosynaptic, glutamate-mediated mechanism [25]. Anandamide acts in the vagal complex at both CB1 and TRPV1 receptors and can affect visceral function by modulating GABA and glutamate release [9,43]. Administration of anandamide or capsaicin into the fourth ventricle increases gastric acid secretion due to TRPV1-modulated modulation of central synaptic mechanisms [44]. Thus, a role for anandamide as an endovanilloid in the vagal complex is supported by the present findings.

Rapid effects of glucocorticoid receptor activation in the hypothalamus has been demonstrated to be mediated by neuron- and perhaps synapse-specific release of retrograde messengers, including anandamide, whose release is stimulated by binding of a putative membrane-associated glucocorticoid receptor [16,37,38,45]. Glucocorticoids rapidly suppress glutamate release in hypothalamic neuroendocrine neurons via a CB1 receptor-dependent mechanism [16] and enhance GABA release through retrograde nitric oxide signaling [38]. Glucocorticoid effects on glutamate release in gastric-related, preautonomic hypothalamic neurons are biphasic, involving both TRPV1 and CB1 receptor activation [18]. We found that DEX, a glucocorticoid receptor agonist, rapidly enhanced GABAergic input to DMV neurons in a concentration-related fashion. The effect was blocked by both spironolactone and mifepristone, consistent with activation of glucocorticoid receptors. We did not differentiate the effect on individual receptors; concentrations of both antagonists may have been high enough in vitro to block either glucocorticoid type I or II receptors. The enhanced mIPSC frequency represents an increase in synaptic GABA release from terminals rather than increased action potential activity in afferent GABA neurons, since sodium channels were continually blocked with TTX. Thus, the enhancement of mIPSC frequency was likely due to effects at the terminals of afferent neurons. The DEX-induced increase in GABA release was insensitive to blockade of CB1 receptors, but was prevented by selective antagonism of TRPV1 receptors with either capsazepine or 5’-iRFT. Thus, the ability of DEX to enhance GABA release depended upon both glucocorticoid receptor binding and TRPV1 receptor activation.

In the DMV, effects on GABA release of exogenously applied TRPV1 agonists like capsaicin were significantly, but not completely, reduced in the presence of glutamate receptor antagonists within five minutes of application. This suggested that TRPV1 agonists bound receptors on glutamatergic terminals to increase glutamate release, which heterosynaptically increased GABAergic synaptic inhibition [25]. Here, we found that DEX increased glutamate release in a sustained fashion and that DEX effects on GABA release were present, but somewhat attenuated, in the presence of ionotropic glutamate receptor blockade. These findings are consistent with the hypothesis that DEX leads to activation of TRPV1 receptors on glutamatergic terminals, resulting in a degree of heterosynaptic enhancement of GABA release in the DMV. Notably, the DEX-induced increase in GABA release was maintained in the presence of kynurenic acid, consistent with additional activation of TRPV1 receptors located on GABAergic terminals. Effects of retrogradely-released endocannabinoids in other brain areas are typically highly localized, resulting in synapse-specific modulation of neurotransmitter release [38,46]. That the effect appeared to be less dependent on heterosynaptic mechanisms than for applied agonists could reflect the highly localized release and function of retrograde messengers.

It is well-established that TRPV1 receptors on terminals of glutamatergic primary viscerosensory vagal afferents modulate vagal terminal activity and also mediate asynchronous glutamate release in the NTS [27–29]. Vagal afferents are also known to synapse on dendrites of DMV neurons in the rat [47]. Further, NTS neurons likely also express TRPV1 message [30], and functional data support the hypothesis that TRPV1 receptors are located on terminals of NTS neurons [25]. While the precise location of TRPV1 receptors that mediate the enhancement of GABA release by DEX in the DMV is uncertain, it is possible that receptors on primary viscerosensory afferents and/or glutamatergic and GABAergic terminals of NTS neurons contacting DMV cells contribute to the response.

The nature of the DEX effect suggested activity at a membrane receptor, similar to that in previous studies of rapid glucocorticoid-induced effects on synaptic input [16]. In support of this, the effect of DEX was mimicked by application of BSA-conjugated DEX, which was assumed to be less able to cross the cell membrane and bind canonical intracellular receptors. In several other brain areas, a membrane-bound glucocorticoid receptor has been proposed, based on electrophysiological or anatomical data [16,19,20]. Similar to hypothalamic neurons, the effect of DEX-induced enhancement of mIPSC frequency in the DMV was prevented when GDP _β_S was used intracellularly to block G protein activity in the recorded neuron. This observation suggests that the effects seen here are secondary to DEX-induced activation of G protein-coupled receptors on the recorded DMV neuron. Together, these data are consistent with the hypothesis that DEX activates a G protein-coupled, membrane-associated receptor on DMV neurons, activation of which leads to a TRPV1-dependent enhancement of GABA release in the DMV.

TRPV1 receptors at synaptic terminals are activated by a number of stimuli, including pH, temperature, and exogenous application of capsaicin or anandamide [25]. In the DMV, CB1 receptor activation diminished synaptic activity moderately over a period of several minutes [26] and after strong depolarization of DMV neurons [31], whereas activation of TRPV1 receptors on presynaptic terminals resulted in a rapid and potent enhancement of GABA release [25]. These effects were recapitulated by anandamide application [25], suggesting that a single endocannabinoid ligand could induce increases and/or decreases in synaptic input. Other retrogradely-released substances have been identified that could act as putative endovanilloids to affect changes in presynaptic terminals [36,43], including in the vagal complex [24]. Several candidates are metabolites of eicosanoid-derived endocannabinoids, including those derived from anandamide [36]. The rapid effects of DEX on TRPV1 receptors seen here could thus be mediated by an anandamide metabolite or more directly by released anandamide or some other related eicosanoid.

Unexpectedly, we did not detect a decrease in synaptic input after DEX application in the presence of TRPV1 antagonists, and blockade of CB1 receptors with AM-251 had little effect on mIPSC frequency or on the DEX-induced increase in GABA release. These data suggest little, if any, CB1 receptor-mediated effect of DEX-induced retrograde messenger release. This could be for several reasons, including that DEX induced the release of an agonist with little activity at CB1 receptors. Another is that DEX-induced activity resulted in low anandamide concentrations; CB1R-mediated effects of applied anandamide are not as robust as are TRPV1-mediated effects at the same concentration [25]. Additionally, the DEX-mediated release of anandamide or other messenger could conceivably occur selectively near synaptic terminals containing TRPV1, but not CB1, receptors. Such synapse-specific release of retrograde messengers by DEX or membrane depolarization has been demonstrated elsewhere in the brain [38,45,48].

Alternatively, the messenger mediating the TRPV1-dependent enhancement of GABA release by DEX could be one of anandamide’s metabolites. The effects of DEX were abrogated by inhibiting activity of FAAH, the enzyme responsible for metabolic processing of anandamide and probably other eicosanoids. Blocking anandamide metabolism might have been expected to increase the extracellular concentration of the agonist, with the expected result that DEX effects would be enhanced. This was not the case; inhibiting FAAH prevented any DEX-induced change in mIPSC frequency. Metabolites of anandamide, including arachidonic acid and it’s derivatives have been previously hypothesized to be candidate ‘endovanilloid’ retrograde messengers [36]. Since preventing metabolism with URB597 prevented the effect of DEX, this suggests the possibility that a metabolite of anandamide (or other endocannabinoid that is metabolized by FAAH) may be the agonist that binds TRPV1 in this system.

Blocking putative anandamide transport with either OMDM-2 or O-2093 might also be expected to increase the agonist effect by preventing reuptake into the recorded DMV cell or nearby neural or glial elements, allowing the agonist to accumulate extracellularly. Notably, the agonist binding site on the TRPV1 receptor is intracellular. Passive or facilitated diffusion or active transport have all been proposed as mechanisms that might allow the agonist passage across the membrane to reach the TRPV1 binding site [36]. In addition to blocking reuptake, blocking transporter function should reduce anandamide transport across the afferent nerve terminal membrane, thereby limiting its access to the intracellular TRPV1 binding site. In support of this mechanism in the DMV, anandamide uptake inhibitors prevented the DEX effect on GABA release.

Endogenous anandamide release results following strong depolarization of DMV neurons, resulting in depolarization-induced suppression of inhibition (i.e., DSI) [31]. Anandamide binding to CB1 receptors in the DMV is proposed as the mediator of that effect, with no detectable TRPV1-mediated activity. The effect of DEX in the present study was affected by inhibition of specific transporters and metabolism of anandamide, suggesting anandamide (or another eicosanoid -derived endocannabinoid with similar mediators of metabolism and transport) as a logical mediator of the mIPSC enhancement by DEX. However, we observed little evidence of CB1 receptor involvement in the response to DEX. It is possible that differences in recording conditions underlie the different responses. For example, the DSI effect observed previously [31] was observed in the presence of a high glucose concentration (i.e., 25 mM), which was more than double that used herein. Prolonged elevation in glucose concentration in mice with type 1 diabetes induces internalization of TRPV1 receptors in the DMV [49], resulting in elimination of their function. If similar receptor internalization resulted during the course of experiments demonstrating DSI, then any TRPV1-mediated effect of depolarization could have been reduced in the presence of the elevated glucose used in those studies. Alternatively, the methods of evoking release (brief depolarization versus DEX application) could result in different pools of endocannabinoid being released and acting on separate afferent terminals [48]. Here, analyses of mIPSCs were performed at a holding potential of -10 mV, which allowed isolation of mIPSCs without the necessity of blocking glutamate receptors, which reduces TRPV1-mediated GABA release [25]. Effects of DEX were evident at this potential, and TPRV1 antagonists alone had no effect on mIPSC frequency, suggesting that continuous depolarization may not have induced continual release of a TRPV1 agonist. Alternatively, TRPV1-mediated effects of tonic depolarization on GABA release may have subsided, leaving a separate DEX-induced release of agonist. Our results are consistent with the hypothesis that DMV cells release anandamide or other related eicosanoid, resulting in a TRPV1-mediated enhancement of GABA release in the DMV in response to DEX, independent of DMV neuron depolarization.

Most DMV neurons are preganglionic parasympathetic neurons that convey information to the digestive system [3]. They tend to exhibit a slow, continuous action potential firing pattern [3], and their activity is heavily modulated by GABAergic synaptic input [4]. Glucocorticoids acting in the CNS are known to affect vagally-mediated metabolic and autonomic functions [9], and effects of glucocorticoids in the DMV include altered blood pressure, decreased emetic responses, and increased gastric acid secretion [10–12,43,44,50]. The effects of DEX on synaptic input to DMV neurons observed here are consistent with many of these effects. The highly regulated feedback facilitation of inhibitory input caused by DEX-induced TRPV1 activation would tend to make DMV cells less susceptible to stimuli that suppress synaptic inhibition and thus stabilize vagal motor output. Loss of this facilitation, as occurs in diabetic animals [49], could significantly influence important autonomic visceral functions. Further study of visceral system-specific effects of DEX activity in the dorsal vagal complex may offer improved understanding of glucocorticoid modulation of autonomic functions, especially those related to gastrointestinal function, digestion, and feeding.
